# Supplementary material for: Baicalin Represses Type Three Secretion System of Pseudomonas aeruginosa through PQS System
Source: Molecules. 2021 Mar 10;26(6):1497. doi: 10.3390/molecules26061497 (PMC8001617; doi:10.3390/molecules26061497)
Supplement: Supplementary file 1 [file molecules-26-01497-s001.pdf]

Table S1. Effect of baicalin on the expression of virulence related genes in *P. aeruginosa*

PAO1

Virulence factors	Relevant characteristics	Effect of DMSO on the expression of genes (Repression/ Induction)
lasI	Autoinducer synthesis protein LasI	_
lasR	AHL dependent transcriptional regulator LasR	_
lasB	elastase synthesis	_
rhlR	AHL dependent transcriptional regulator RhlR	_
rhlI	Autoinducer synthesis protein RhlI	_
phzA1	Phenazine biosynthesis protein	+
phzA2	Phenazine biosynthesis protein	+
pqsA	Anthranilate-coenzyme A ligase.	_
pqsR	Transcriptional regulator PqsR	_
pilG	Twitching motility protein PilG	_
fliC	Flagellin type B	_
exoS	Exoenzyme S (ADP-ribosyltransferase)	_
exoY	Adenylate cyclase ExoY	_
exoT	Exoenzyme T	_
exsD	Transcriptional anti-activator of the type III secretion system ExsD	_
exsC	Exoenzyme S synthesis protein C precursor.	_
oprH	Outer membrane protein H (OprH)	_
xcpR	General secretion pathway protein E	_
migA	Alpha-1,6-rhamnosyltransferase MigA	_
aprA	Alkaline metalloproteinase precursor	_
rnr	Exoribonuclease RNase R	_
gacA	Response regulator GacA	_
rpoS	Sigma factor RpoS	_
vfr	Transcriptional regulator Vfr	_
rsmA	carbon storage regulator RsmA	_
rsmY	Regulatory RNA RsmY	_
rsmZ	Regulatory RNA RsmZ	_

"_" repression on gene expression by baicalin; "+" enhancement on gene expression by baicalin.
